# Change of Direction Assessment Following Anterior Cruciate Ligament Reconstruction: A Review of Current Practice and Considerations to Enhance Practical Application

**DOI:** 10.1007/s40279-019-01189-4

**Published:** 2019-09-17

**Authors:** Joao Beleboni Marques, Darren James Paul, Phil Graham-Smith, Paul James Read

**Affiliations:** 1 Aspetar, Orthopaedic and Sports Medicine Hospital, Sports City Street, Al Waab, Beverly Hills Garden 14, Villa 30, P.O. Box 29222, Doha, Qatar; 2 Aspire Academy, Sports City Street, P.O. Box 29222, Doha, Qatar

## Abstract

Change of direction (CoD) has been indicated as a key mechanism in the occurrence of anterior cruciate ligament (ACL) injury during invasion sports. Despite these associations, assessments of knee function in athletic populations at the time of return to sport following ACL reconstruction (ACLr) have often focused on strength and single-leg hop tests, with a paucity of evidence to describe the CoD characteristics. Therefore, the aim of this narrative review was to describe the movement strategies exhibited following ACLr during CoD tasks and to critically analyze the range of tests that have been used. Specifically, we examined their ability to identify between-limb deficits and individuals who display a heightened risk of secondary injury and/or reductions in their level of pre-injury performance. MEDLINE, PubMed and SPORT Discuss databases were used and 13 articles were identified that met the inclusion criteria. Examination of the available literature indicates that current field-based practices are not representative of relevant sport demands and are unable to effectively assess knee function following ACLr. Laboratory-based studies have identified residual deficits and altered movement strategies at the time of return to sport, and this in part may be related to risk of re-injury. However, these assessments exhibit inherent limitations and are not practically viable for monitoring progress during rehabilitation. Consequently, alternative solutions that are more-aligned with the multitude of factors occurring during CoD maneuvers in chaotic sports environments are warranted to allow practitioners to ‘bridge the gap’ between the laboratory and the sports field/court. This approach may facilitate a more informed decision-making process with the end goal being, a heightened ‘return to performance’ and a lower risk of re-injury.

## Key points

**Table:** 

CoD tasks have been recognized as a key mechanism of non-contact ACL injury. However, there is currently a lack of research examining CoD tests as a means to assess knee function following ACLr and associations with secondary injuries or a return to pre-injury levels of performance.
Existing literature offers a combination of field and laboratory-based CoD tests. Laboratory-based assessments appear to be more sensitive in their ability to identify alterations in movement mechanics following ACLr but they also lack ecological validity and are currently not practically viable to systematically assess knee function during the rehabilitation process.
Practitioners are encouraged to develop practically viable solutions to bridge the gap between laboratory and sports environment, while considering relevant conditions (e.g., planned vs. unplanned, fatigue state) and constraints (e.g., cutting angle, approach velocity). This approach can enhance the RTS decision.

## Introduction

The anterior cruciate ligament (ACL) is commonly injured during sports participation, particularly those involving jumping, pivoting, cutting or change of direction (CoD) maneuvers [32]. ACL-deficiency is suggested to cause knee instability and changes in motor control strategy (i.e., proprioception, postural control, muscle strength, movement and recruitment pattern) [14]. Thus, athletes who wish to continue in hard cutting and pivoting sports after ACL injury are often advised to undergo ACL reconstruction (ACLr) to re-establish mechanical knee stability [52]. However, statistics show that 35% of athletes after ACLr do not return to pre-injury sports level within 2 years [4]. Moreover, up to 20% of those returning to sport in the first year from surgery experience a second ACL rupture [85].

Evidence suggests criterion-based rehabilitation and return to sport (RTS) progressions should be evaluated with appropriate tests of impairments, activities, participation and contextual factors and combined with a minimum 9-month time criterion [9]. The 2016 consensus on RTS [5] outlines five recommendations to guide the choice of RTS tests: (1) use a group of tests (test battery). (2) Choose open tasks (less controlled) over closed tasks (more controlled) where possible. (3) Include tests with reactive decision-making elements. (4) Assess psychological readiness to return to sport. (5) Monitor internal and external workload. However, a recent scoping review indicates that published research on RTS often does not mirror the 2016 consensus statement [9]. The efficacy of current ‘evidenced based’ practice in the area of return to sport assessment has also been questioned, with athletes passing specified criteria reducing the risk of subsequent graft rupture by 60%, but this was combined with 235% increased risk of contralateral graft rupture [84]. Thus, exploration of other factors that contribute to reductions in overall ACL re-injury risk is warranted.

CoD has been recognized as a mechanism of non-contact ACL injury [53, 64, 82]; however, there is a distinct lack of research pertaining to COD performance as component of return to sport testing and the utility of these assessments to identify associations with secondary injuries or a return to pre-injury levels of competition and performance. The aim of this narrative review is to examine the available literature pertaining to CoD assessment following ACLr to determine their suitability as a readiness to RTS tool. In addition, key considerations are provided to aid in the design of CoD assessment, in an attempt to optimize their use as part of the end stage rehabilitation and return to sport process.

## Methodology

The lead author conducted computer searches of MEDLINE, PubMed and SPORT Discuss electronic databases for studies pertaining to CoD assessments following ACLr published between 1970 and April 2109. The search strategy chose to combine specific terms with the words ‘change of direction’ and ‘anterior cruciate ligament reconstruction’ to ensure relevant articles were extracted. This included: ‘change of direction test following anterior cruciate ligament reconstruction’, ‘change of direction assessment following anterior cruciate ligament reconstruction’, ‘functional assessments following anterior cruciate ligament reconstruction’, ‘assessment of knee function following anterior cruciate ligament reconstruction’, ‘return to sport criteria following anterior cruciate ligament reconstruction’, ‘knee mechanics during change of direction following anterior cruciate ligament reconstruction’. Studies were deemed relevant after scanning the title and abstract and where subsequent access to the full text was available. The reference lists of each study were also examined to ensure no further articles were omitted from the search process. Inclusion in this review was based on:

- Study design: original articles that describe CoD characteristics of patients following ACLr published in English language. Systematic reviews, conference abstracts, case studies, narrative reviews and non-peer reviewed studies were excluded.
- Participants: male and female patients at any age or activity level (e.g., athletes or non-athletes) who had undergone primary ACLr with an autograft (i.e., hamstring or bone-patellar tendon-bone) or allograft surgery techniques. Studies that have not reported ACLr as the primary procedure performed were excluded.
- Outcomes: any measure or index adopted to describe CoD performance and/or movement characteristics following ACLr.
- Time: all postsurgical time frames were included.

## Change of Direction as a Risk Factor for ACL Injury

A change of direction is characterized as the “skills and abilities needed to change movement direction, velocity, or modes” [22]. It is a highly demanding task recognized as a key mechanism of non-contact ACL injury [53, 64, 82]. External knee valgus moment, internal rotation moment and knee flexion angles are considered to be movement patterns that are causative factors of ACL injuries during CoD [49, 55, 64]. While this may be the mechanical profile of how an ACL injury occurs, there are also many additional factors relating to the execution of CoD tasks that contribute to increased risk. For example, evidence shows ACL injury incidence may be increased during; competition compared to training [79], the latter stages of competition [6], unanticipated reactive tasks [46], close proximity to an opponent [12], defending [12], as well as attacking [53], visually distracted situations [26], when carrying equipment [18] and/or dual tasks where attention is divided [3]. This denotes that the occurrence of an ACL injury is complex and there are many possible nuances that may result in this catastrophic outcome.

## Change of Direction Following ACL Reconstruction

Change of Direction tests have been less commonly applied during ACL assessment protocols compared to other tests including strength and hop testing [9]. This approach provides some indication of an individual physical capacity; however, these tests alone may provide an incomplete evaluation of an athlete functional capability following ACLr [57]. Existing literature has used a combination of field and lab-based tests to assess knee function following ACLr and to determine readiness to RTS (Table 1).

**Table 1:** Studies that have adopted CoD tests to assess knee function following ACLr

Study	Subjects/period of assessment	Aim	Testing protocol/measurement method	Main results
Clark et al. [19]	Ten male athletes from Division I University with ACLr; 18–30 years; assessed after full clearance to sports participation (> 9-month post-surgery)	Comparison between involved and uninvolved limb	90° cutting; eight-camera 3-D motion analysis	A 5° measurement of error off of neutral was utilized as reference to indicate meaningful kinematics changes. 80% of the athletes demonstrated significant knee valgus (> 5°) of the involved knee during the cutting task. However, significant knee valgus (> 5°) was also observed in the uninvolved knee for 60% of the athletes. This results question whether returning an involved limb to the standard of the uninvolved limb should be considered as gold standard
King et al. [41]	156 male athletes with ACLr from multidirectional sports and 62 healthy control; 18–35 years; assessed 9-month after surgery	Examine differences in asymmetry of biomechanical and performance variables during PLA and UNP CoD testing between ACLr athletes and matched healthy cohort	90° cutting; eight-camera motion analysis system, two force platforms and light timing system	There was a significant difference in asymmetry of CoD times between groups for both PLA (*p* = 0.004) and UNP (*p* = 0.008) conditions, with greater asymmetry in the ACLr group compared with healthy matched control; however, the magnitude of the difference had a small effect size (0.4). During PLA CoD, ACLr group exhibited greater asymmetry in vertical, medial and posterior GRF as well as greater asymmetry for hip abduction moment after initial contact compared to healthy control group. During UNP CoD, ACLr group exhibited greater asymmetry in vertical and medial GRF as well as knee flexion angle compared to healthy control group. However, there was greater asymmetry in the healthy control group for trunk-on-pelvis flexion angle. The ACLr group was more asymmetrical compared to healthy counterparts, suggesting incomplete restoration of normal movement 9 months after ACLr
King et al. [42]	156 male athletes (Gaelic Football, Soccer, Hurling, Rugby) with ACLr; 18–35 years; assessed 9-month after surgery	Comparison between involved and uninvolved limb and between PLA and UNP conditions	90° cutting; eight-camera motion analysis system, two force platforms and light timing system	Involved limb exhibited different biomechanical responses (e.g., less knee flexion/extension moment, knee internal/external rotation moments) compared to uninvolved limb during both PLA and UNP condition. Unplanned CoD elicited less contralateral pelvis rotation, distance from center of mass to the ankle in frontal plane, posterior GRF and greater hip abduction compared to PLA. No changes in time to complete the CoD testing were observed for both involved and uninvolved limb and between PLA and UNP conditions, suggesting that performance-based criteria may not be the most sensitive criterion to discharge ACLr patients back to sports participation
Kyritsis et al. [44]	158 male professional athletes (Football, Handball, others) with ACLr; 22.0 ± 5.0 years; assessed at the end of rehabilitation prior to discharge	Evaluate discharge criteria and its association with ACL re-injury after RTS	Standardized *t* test amongst others (e.g., strength, hop); Stopwatch	A cut-off of < 11 s during *t* test was established as CoD criterion to discharge patients back to sports participation. There was no association with future injury risk as an independent test but formed part of a testing battery where completion resulted in lower re-injury risk
Jang et al. [34]	67 male athletes (Football, Basketball, Volleyball, others) with ACLr. RTS (*n *= 51, 21.9 ± 4.0 years) and non-RTS (*n *= 16, 21.8 ± 3.5 years); assessed at 6, 12, 24, and 36-month after surgery	Comparison between RTS and non-RTS groups	Co-contraction, Carioca, and Shuttle run tests; Stopwatch	The RTS group exhibited higher performance in co-contraction (RTS = 14.2 ± 1.4 vs. non-RTS = 15.8 ± 2.2, *p* = 0.010) and carioca (RTS = 8.5 ± 1.5 vs. non-RTS = 9.9 ± 2.6, *p* = 0.045) tests compared to non-RTS counterparts, suggesting that these tests may assess rotational stability on the ACLr knee. No differences in performance (RTS = 7.5 ± 0.5 vs. non-RTS = 7.8 ± 1.2, *p* = 0.607) was found between groups for the shuttle run test
Pollard et al. [67]	20 female soccer players with ACLr (*n *= 10, 23.2 ± 3.4 years) and Healthy control (*n *= 10, 21.0 ± 1.2 years); assessed 12-month after surgery	Comparison between ACLr and control groups	45° sidestep cutting; Reflective markers (14-mm spheres) with 3-D motion analysis system and floor-embedded force platform	The ACLr players exhibited increased lower extremity variability during the cutting task as compared with the healthy counterparts in the following couplings: hip rotation/knee abduction-adduction (ACLr = 27.2° ± 11.5° vs. control = 19.7° ± 6.8°, *p* = 0.04); hip flexion-extension/knee abduction-adduction (ACLr = 26.0° ± 13.3° vs. control = 18.6° ± 5.3°, *p* = 0.05); knee abduction-adduction/knee flexion-extension (ACLr = 13.5° ± 5.7° vs. control = 7.3° ± 2.7°, *p* < 0.01); and knee abduction-adduction/knee rotation (ACLr = 26.4° ± 10.8° vs. control = 19.3° ± 4.5°, *p* = 0.03). Increased movement variability during CoD task is likely reflective of altered neuromuscular control as a result of ACLr
Stearns et al. [78]	24 female soccer players with ACLr (*n* = 12, 23.7 ± 1.9 years) and Healthy control (n=12, 21.3 ± 1.2 years); assessed 12-month after surgery	Comparison between ACLr and control groups	Sidestep cutting; 8-camera 3D motion analysis system and floor-embedded force platform	The ACLr group exhibited increased mean knee abduction angles (ACLr = 3.8° vs. control = 1.8°, *p *= 0.03), and peak knee adductor moments (ACLr = 1.33 N.m/kg vs. control = 0.80 N.m/kg, *p *= 0.004) compared with healthy matched control, suggesting higher risk of re-injury upon RTS participation
Kong et al. [43]	60 male patients with ACLr (*n *= 30, 23.4 ± 3.1 years) and Healthy control (n=30, 24.7 ± 2.1 years); assessed 6-month after surgery	Reliability in the heathy group and correlation between tests in ACLR group	Co-contraction, Carioca, and Shuttle run tests; Stopwatch	High test–retest correlation values were found for co-contraction (*r *= 0.511, *p *= 0.025), shuttle run (*r *= 0.746, *p *= 0.000), and carioca (*r *= 0.742, *p *= 0.000) tests. High correlation was also found between the three tests and strength tests (Isokinetic, hop). ACLr group exhibited lower performance compared to healthy match controls in all the three tests (co-contraction: ACLr = 1.89 ± 2.91 vs. control = 13.3 ± 1.04; shuttle run: ACLr = 7.67 ± 0.97 vs. control = 6.49 ± 0.39; and carioca: ACLr = 9.31 ± 2.43 vs. control = 6.96 ± 0.81)
Myer et al. [56]	36 athletes (Football, Soccer, Basketball, Volleyball) with ACLr (n=18, 16.9 ± 2.1 years) and Heathy control (n=20, 16.9 ± 1.1 years); assessed 12-month after surgery	Test side-to-side symmetry	Modified t-test; Stopwatch	No asymmetries were identified in modified t-test for ACLr group post-surgery. Also LSI was not different between ACLr group and healthy matched control (*p* > 0.05). This finding opposed the hop tests, which had heightened sensitivity and displayed different LSI scores between groups (ACLr = 92% vs. control = 100%, *p* < 0.001). The results suggest that the modified version of t-test was not sensitive to identify deficiencies between involved and uninvolved limb
Keays et al. [39]	31 patients with ACLr Male (*n *= 22) Female (*n *= 9) 19–38 years (mean = 27 years); assessed 6-month after surgery	Correlation between isokinetic strength test and shuttle run, carioca and side step tests	Shuttle run, Carioca, and Side step tests; Cybex Isokinetic Dynamometer and Stopwatch	Correlation values were found between shuttle run (*r *= 0.498, *p *= 0.004), carioca (*r *= 0.474, *p *= 0.007), side step (*r *= 0.528, *p *= 0.002) tests and isokinetic quadriceps strength test 6 months after ACLr. These post-surgery correlation values were higher compared to pre-surgery. No correlations were found between shuttle run, side step and carioca tests and isokinetic hamstring strength test
Keays et al. [38]	31 patients with ACLr Male (*n *= 22) Female (*n *= 9) 19 – 38 years (mean = 27 years); assessed 6-month after surgery	Comparison between involved and uninvolved limb	Shuttle run, Carioca, and Side step tests; Stopwatch	A 10, 17, and 23% improvement in performance were found for Shuttle run (pre-surgery = 9.83 ± 1.46 s vs. post-surgery = 8.86 ± 1.04 s, *p* < 0.01), Side step (pre-surgery = 13.03 ± 3.48 s vs. post-surgery = 10.86 ± 1.28 s, *p* < 0.01), and Carioca (pre-surgery = 17.42 ± 5.62 s vs. post-surgery = 13.38 ± 2.23 s, *p* < 0.001) tests, respectively, 6-month following ACLr
Lephart et al. [48]	41 patients with ACLr Male (*n *= 32) Female (*n *= 9) 19–38 years (mean = 22.7 years); assessed within 10–36 months’ post-surgery	Performance comparison between RTS (*n *= 29) and Non-RTS (*n *= 12) groups	Co-contraction, Carioca, and Shuttle run tests; Stopwatch	The RTS group exhibited better performance during the shuttle run (7.45 ± 8.2 s vs. non-RTS group = 9.67 ± 3.18 s, *p* < 0.01), carioca (8.54 ± 1.93 s vs. non-RTS group = 17.31 ± 14.33 s, *p* < 0.01), and co-contraction (14.96 ± 4.48 s vs. non-RTS group = 20.70 ± 12.42 s, *p* < 0.05) tests compared to non-RTS counterparts
Tibone et al. [80]	11 patients with ACLr Male (*n *= 10) Female (*n *= 1) 18–45 years (mean = 25.5 years); assessed 2-years after surgery	Comparison between involved and uninvolved limb	Straight cut and Cross cut maneuvers; Force plate platform	No differences in cutting index were found between involved and uninvolved limb for both CoD tasks (straight cut: involved = 1348.7 ± 1573.69 vs. uninvolved = 1898.8 ± 2633.65, *p *= NS; cross cut: involved = 440.5 ± 476.04 vs. uninvolved = 421.7 ± 279.01, *p *= NS), suggesting mechanical responses were normalized 2-years after surgery

*ACL* anterior cruciate ligament, *ACLr* anterior cruciate ligament reconstruction, *n* number, *CoD* change of direction, *PLA* planned, *UNP* unplanned, *RTS* return to sport, *LSI* limb symmetry index, *NS* non-significant

### Field-Based Assessments

**Fig. 1:**
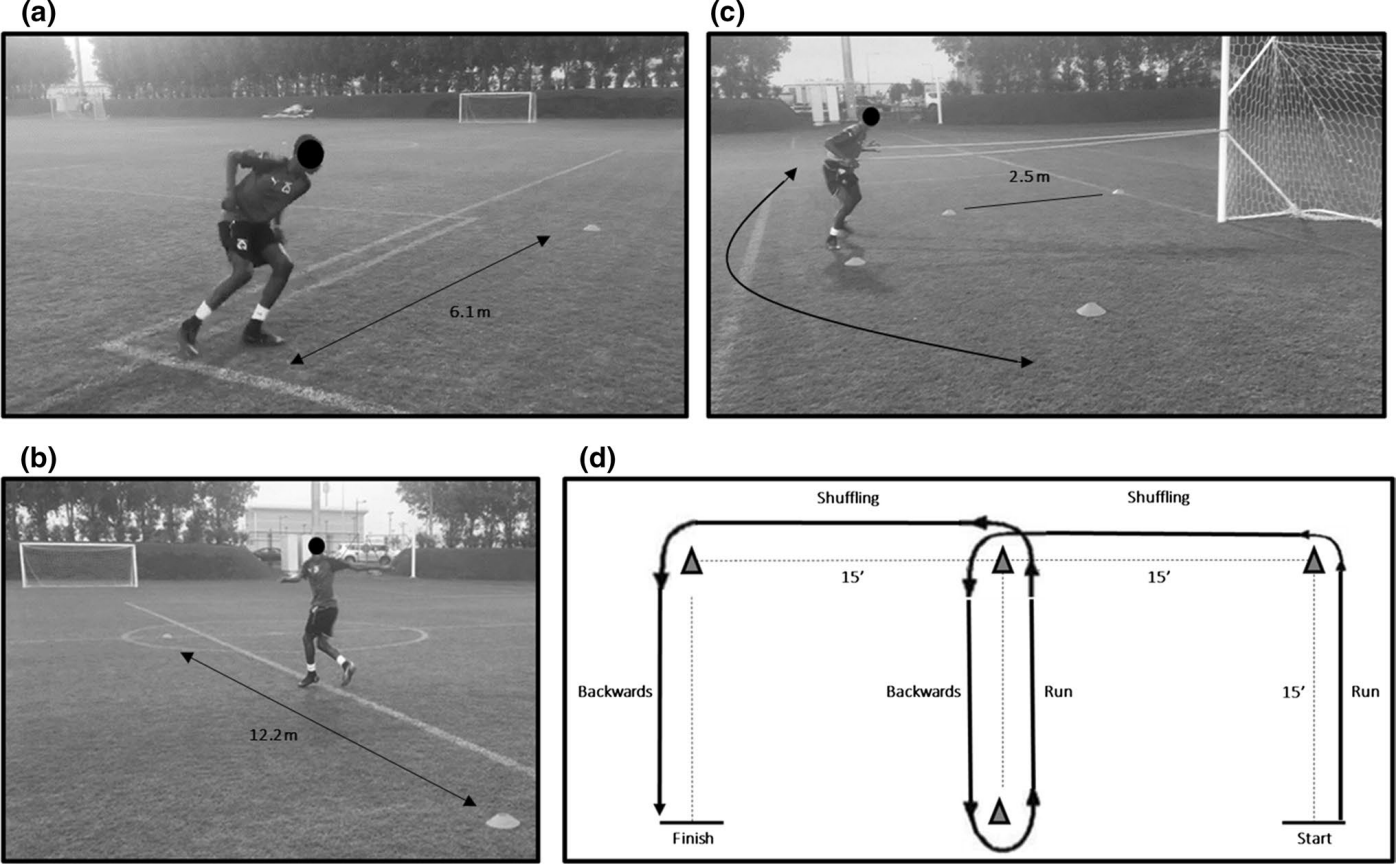
**a**–**d** Illustration of the functional performance tests used to assess knee function following ACLR. **a** Shuttle run test (adapted from Jang et al. [34]). The athlete performs four lengths of 6.1 m each to complete 24.4 m in the shortest amount of time possible, reversing direction after the completion of each length; **b** carioca test (adapted from Jang et al. [34]). Using an alternating crossover step, the subject moves laterally to the right 12.2 m, then reverses direction to return to the starting position; **c** co-contraction test (adapted from Jang et al. [34]). The patient moves in a side step or shuffle fashion around the periphery of a 2.5 m radius semicircle. The test is complete when five semicircle lengths have been performed; **d** modified *t* test (adapted from Myer et al. [56]). The test requires a combination of 15 ft of forward running, shuffling and backwards movement to the left side and right sides

Three field-based testing protocols have commonly been adopted [34, 38, 39, 43, 48]. These includes the shuttle run, co-contraction, and carioca tests. The shuttle run test (Fig. 1a) has been suggested to reproduce acceleration and deceleration forces that are common in sports activities [43]; the co-contraction test (Fig. 1b) imparts rotational forces on the knee that cause tibial translation and are mostly controlled by thigh musculature; while the carioca test (Fig. 1c) reproduces the pivot-shift phenomenon in the ACL-deficient knee when subjects move laterally with a cross-over step [43].

Kong and colleagues [43] reported moderate to high test re-test reliability (*r *= 0.51–0.74) of all three of the aforementioned CoD assessments (shuttle run, co-contraction and carioca tests) as well as their relationships with isokinetic strength (*r* = 0.45–0.52) and one leg hop for distance tests (*r* = 0.59–0.75) on both the involved and uninvolved limbs. These data are supported by earlier research which showed similar relationships (*r* = 0.46–0.53, *p* < 0.05) between these tests and quadriceps isokinetic strength 6 months after ACLr [38]. However, it should be acknowledged that the isokinetic testing modes used were concentric only [38, 43], and eccentric modes of contraction (for both the quadriceps and hamstrings) may be more relevant to CoD tasks. Other studies advocated that these tests were sensitive to distinguish athletes’ readiness to return to sports activity [34, 48]. Keays et al. [38] observed that ACLr patients improved their time in the shuttle run, co-contraction and carioca tests by 10, 17, and 23%, respectively, at 6-month post-surgery compared to pre-surgery values. According to Lephart et al. [48] and Jang et al. [34], better performance was found amongst shuttle run, co-contraction and carioca tests for the patients that returned to sports within 1–2 years after ACLr compared to those who did not return.

Most recently, Kyritsis et al. [44] used the *T* test with a performance-based cut-off pass criterion of < 11 s as part of a RTS testing battery to identify athletes’ who sustained a secondary ACL rupture [44]. No associations were shown between performance on this test and risk of future ACL injury; however, athletes who did not pass the entire battery of tests (including isokinetic strength and 3 single hop tests), had a four times greater risk of sustaining a second ACL injury compared to those who met the full criteria [44]. Myer et al. [56] proposed a modified version of the *T* test (Fig. 1d) to examine if differences between involved and un-involved limbs (by providing alternate directions of travel) could be identified in athletes following ACLr. Similarly, this approach was unable to identify differences between limbs, with the authors indicating the length of the test and the inherent repeated bilateral nature of the task with multiple contacts on each foot mask deficits of the involved lower extremity.

### Laboratory Assessments

Stearns and Pollard [78] compared the kinematics and joint kinetics of female soccer players with a history of ACL reconstruction vs. healthy matched controls during a 45° sidestep cutting maneuver. The assessment was performed after players were cleared to return to full sports participation (> 9-month post ACLr). The authors found that players with a history of ACLr displayed increased knee abduction angles and knee adductor moments compared with healthy players. This finding is consistent with the proposed mechanism of increased loading of the ACL and hence, may predispose athletes to a greater risk of re-injury. Using the same CoD task (45° sidestep cutting), Pollard et al. [67] examined lower extremity coupling variability in female soccer players 12-month post ACLr. The authors considered this approach as an alternative to examine joint interactions and the variability of these interactions (i.e., neuromuscular control) during a complex movement such as CoD. It has been reported that ACLr players exhibited increased lower extremity variability as compared with the healthy counterparts in the following couplings: hip rotation/knee abduction-adduction, hip flexion–extension/knee abduction-adduction, knee abduction-adduction/knee flexion-extension, and knee abduction-adduction/knee rotation, suggesting a compromised neuromuscular control as a result of ACLr. This impairment in controlling joint movements during CoD may predispose the players to re-injury upon RTS participation given the higher environmental demands of the sport. Earlier work by Tibone and Antich [80] reported that a cutting index (defined by the authors as the product of the medial-lateral, anterior–posterior, and vertical forces and the angle of cut, divided by the product of the approach time and the time spent on the force plate) of a cross cutting task was normalized 2 years after ACLr.

Recently, King et al. [41] examined the performance and biomechanics of a 90° cutting task in both planned and unplanned (light stimuli) conditions. They tested 156 athletes from different sports background (e.g., Gaelic football, soccer, rugby) 9 months after ACLr. The authors showed different biomechanical responses through the kinetic chain between involved and uninvolved limbs during both CoD conditions (planned and unplanned) despite no differences in CoD performance time. The involved limb displayed compensatory mechanisms; specifically, less knee flexion and less knee extension moment in the sagittal plane, and lower knee valgus moment, ankle external rotation moment and knee internal rotation angle and external rotation moment in the frontal/transverse planes. The authors also reported biomechanical differences between planned and unplanned CoDs in variables that have been previously associated with the mechanism of ACL injury (i.e., less contralateral pelvis rotation, distance from center of mass to the ankle in frontal plane, posterior GRF and greater hip abduction) during unplanned cutting. However, similar biomechanical differences have been identified between planned and unplanned CoD tasks in non-injured athletes [7]. Therefore, it is unknown whether the biomechanical differences found by King et al. [41] were due to deficits following ACLr or to the nature of the task constraints.

In a later analysis, King et al. [42] reported the differences in magnitude of asymmetry of biomechanical and performance variables between the ACLr group and healthy matched controls. They found a significant difference in asymmetry of CoD times between groups for both CoD conditions (planned and unplanned), with greater asymmetry in the ACLr group compared with athletes with no history of ACL injury; however, small magnitude of the difference was reported. For instance, the ACLr group exhibited greater asymmetry in vertical, medial and posterior ground reaction force (GRF) as well as greater asymmetry for hip abduction moment after initial contact compared to control group during planned CoD. For the CoD task under unplanned condition, the ACLr group exhibited greater asymmetry in vertical and medial GRF as well as knee flexion angle compared to healthy matched control. However, there was greater asymmetry in the healthy control group for trunk-on-pelvis flexion angle. These findings suggested that the ACLr group was more asymmetrical compared to healthy counterparts, indicating an incomplete restoration of normal movement 9 months after ACLr.

Using the same CoD task (90° cutting), Clark [19] examined frontal plane knee kinematics in university athletes that have fully returned to sports participation following ACLr. The authors found that 8 out of 10 athletes (80%) exhibited ≥ 5° (established as meaningful index value) of knee valgus angle on the involved limb during the CoD task. Interestingly, the authors also reported that 60% of the uninvolved lower extremities demonstrated a substantial valgus angle as well, suggesting that asymmetries may be inherent to the demands of the CoD task independently of the limb examined. These findings question whether returning an involved limb to the standard of the uninvolved limb should be considered as gold standard metric to discharge patients back to sports participation.

### Critical Overview of CoD Assessment Used Following ACLr

In the aforementioned literature, field-based studies have adopted different assessment methods that vary from no changes in direction (e.g., co-contraction, carioca), to 2 (e.g., shuttle run) or 6 directional changes (e.g., agility *t* test, modified *t* test). Such variation would elicit different energetic requirement and diverse responses of the neuromuscular system; thus, these tests are likely independent from one another [13]. The co-contraction, carioca and shuttle run tests have been recommended by previous studies to inform RTS decision-making [34, 38, 39, 43, 48]. However, it is important to critically appraise their use as CoD tests. Specifically, the movement patterns displayed bears little to no resemblance of how an athlete would move in a sport-specific setting, particularly during cutting and turning maneuvers. CoD at high speed requires an individual to rapidly decelerate to change their state of momentum, and then re-direct their body towards the intended direction of travel prior to re-accelerating with minimal time loss [73]. From these tests, it would appear that the shuttle run test most closely resembles the components of a CoD assessment. However, studies that have adopted this testing protocol have only used completion time (i.e., performance) as a metric to evaluate athletes’ readiness which does not examine the strategies used to execute each CoD. It should be noted that during this type of task (i.e., 180° turn), the large majority of the time (70%) is influenced by linear speed with a substantially lower proportion (30%) spent during the turning phase [60], and this may mask the actual CoD performance of an athlete [62]. As a result, this approach may not be adequate to identify deficits in movement, particularly between involved and uninvolved limbs.

A method which has been developed to overcome these limitations and isolate the CoD aspect of the task is the CoD deficit [61]. This measure is easily obtained by performing a linear sprint matching the same distance shown during the test and then subtracting this value from the total CoD time (e.g., CoD deficit = 5–0–5 time—10 m sprint time) and may more accurately delineate individuals with better CoD speed. However, this method has yet to be validated for use in athletes following ACLr. In addition, evaluating the entry and exit velocity may be of interest to more clearly elucidate how the direction change is performed, further removing the effect of confounding factors such as linear speed [62].

In a final point of consideration is that no differences in performance (time to complete the task) scores were frequently observed across the broad range of running based testing protocols in-spite of biomechanical deficits. While laboratory assessments provide some diagnostic value and are useful and help us to better understand the mechanics of CoD, this approach is time consuming, laborious and lacking ecological validity. Practically, sports teams may not have access to these resources and/or may not have the funds to access them. Therefore, consideration of a wider range of contextual factors is needed to develop protocols which are more sensitive to identify deficits in task execution. In addition, the increase in use of micro portable technology [58] may be a more practically viable and cost-effective approach but further research is needed to examine their utility for the assessment of CoD in athletes return to sport following ACLr.

## Assessment Considerations

### Competitive vs. Non-competitive

The essence of all sports is competition. Movements that occur in a laboratory environment will likely be different from movements in real game situations. Performing testing in the laboratory environment is easier to control experimentally but does not consider the presence of opponents and teammates [45, 55]. While this area has not been examined in athletes following ACLr, exploration may offer an insight into the strategies applied in different sporting contexts that more closely resemble the environments in which they compete and excel. For instance, basketball players were shown to have significantly better reaction time performing against a competitor than by themselves (690.6 ± 83.8 ms vs. 805.8 ± 101.1 ms, respectively) [89]. Such findings may infer that patients/athletes may not perform ‘maximally’ during traditional testing when performed alone and the task constraints and test stimuli do not allow elite performers to best utilize their heightened perceptual-cognitive abilities. The inclusion of an opponent/competitor may offer a more appropriate stimulus during which to examine both the athlete’s performance and mechanics used. However, this approach is more susceptible to reduced task reliability; thus, further research is warranted to develop a range of CoD assessment modes using human stimuli and examine their ability to provide reproducible and reliable results that can be used to help monitor the athletes return to sport. In addition, virtual reality simulation may provide a suitable competitive environment with a range of scenarios, offering some element of control in a chaotic environment to improve measurement consistency. However, this again requires further investigation, and practically viability should be at the forefront of future developments in this area.

### Planned vs. Unplanned

Rarely in match play do rapid direction changes take place in optimal conditions where athletes have time to select the appropriate movement strategy. Risk factors for ACL injury are also higher during unplanned CoD maneuvers due to the greater external load and mechanical stress placed at the knee joint [7]. Planned CoDs that have been used previously in the assessment of CoD following ACLr (i.e., carioca, shuttle, co contraction, *T* test, cutting) without temporal constraints may afford sufficient time for the adoption of a ‘safer’ and more optimal movement execution. For example, the support foot placement strategy (i.e., more medial to the pelvic midline) prior to initial contact of the push-off foot to initiate the direction change, thus lowering the mechanical stress on the knee [47]. Performing an unplanned task can be in response to either generic (react against 2 dimension planned and unplanned light-based directional arrows) and quasi-realistic (react against 1 or 2 defenders’ scenarios in a 3-dimension video projection) external stimuli. However, the generic unplanned condition (i.e., reacting to a light system) utilized during the CoD test does not provide an ecologically valid stimulus. It is important to highlight as well that this type of generic (light) stimulus does not resemble those that frequently are present in sport to distinguish between elite and sub-elite performers [73]. High-performance athletes will use their “game knowledge” and anticipate situations based on phase of play sequences and then react to the kinematic cues displayed by their opponents [1, 2, 25]. Therefore, using tests that involve generic cues (such as light stimulus) are likely to be limited in their ability to assess transferrable sports specific abilities of athletes which require integration of perception-action coupling, and decision-making to effectively execute a CoD task. This lack of realistic scenario may limit our ability to fully understand the impact of a true un-planned ‘agility’ action on ACL injury risk and this point is further highlighted by recent research.

### Fatigued vs. Non-fatigued

Change of direction assessments are typically conducted when athletes are in a fully recovered state. In most sports, fatigue and agility tasks rarely exist independent of one another; thus, their combined manifestation may present as a worst case scenario in terms of injury risk. Studies have attempted to examine fatigue-induced changes in lower extremity mechanics and their association with ACL injury risk following prolonged activities. For instance, Zebis et al. [88], found that muscle fatigue induced by a simulated handball game elicited alterations in neuromuscular responses. A large reduction in ACL-agonist muscle (i.e., hamstring) activity has been observed during a side cutting movement following a preceding fatigue protocol (requested participants to perform sidestep and cross-over maneuvers, low- to high-intensity running and sprinting intermittently during 50 min), decreasing dynamic knee stability at initial contact and this might expose athletes to higher risk of ACL injury [87]. Savage et al. [69] also found larger knee joint angles and knee extension and internal rotation moments during a sidestep cutting task following a prolonged running protocol that simulated a game-like activity (e.g., walk, jog, fast run and maximal sprint). The impact of fatigue on movement pattern has not been exclusively identified through prolonged activities. According to Cortes et al. [17], a short-term (e.g., 6-min) fatigue testing protocol can be sufficient to induce several biomechanical changes in the lower extremity (i.e., decreased knee and hip flexion and increased knee internal rotation moments) during an unplanned sidestep cutting. Although the available evidence does not indicate a clear fatigue mechanism, it is apparent that neuromuscular control is altered when athletes are exposed to either competition [30] and/or protocols designed to elicit fatigue [17]. As a result, it may be relevant to implement CoD testing following short-term (e.g., repeated sprints) or long-term (e.g., simulated game activity) fatigue testing protocols to assess ACLr patients’ readiness. This approach allows practitioners to identify the ability of the patient to maintain a safer movement pattern during CoD while fatigued.

## Assessment Characteristics: Task and Environment Constraints

### Cutting Angle

**Fig. 2:**
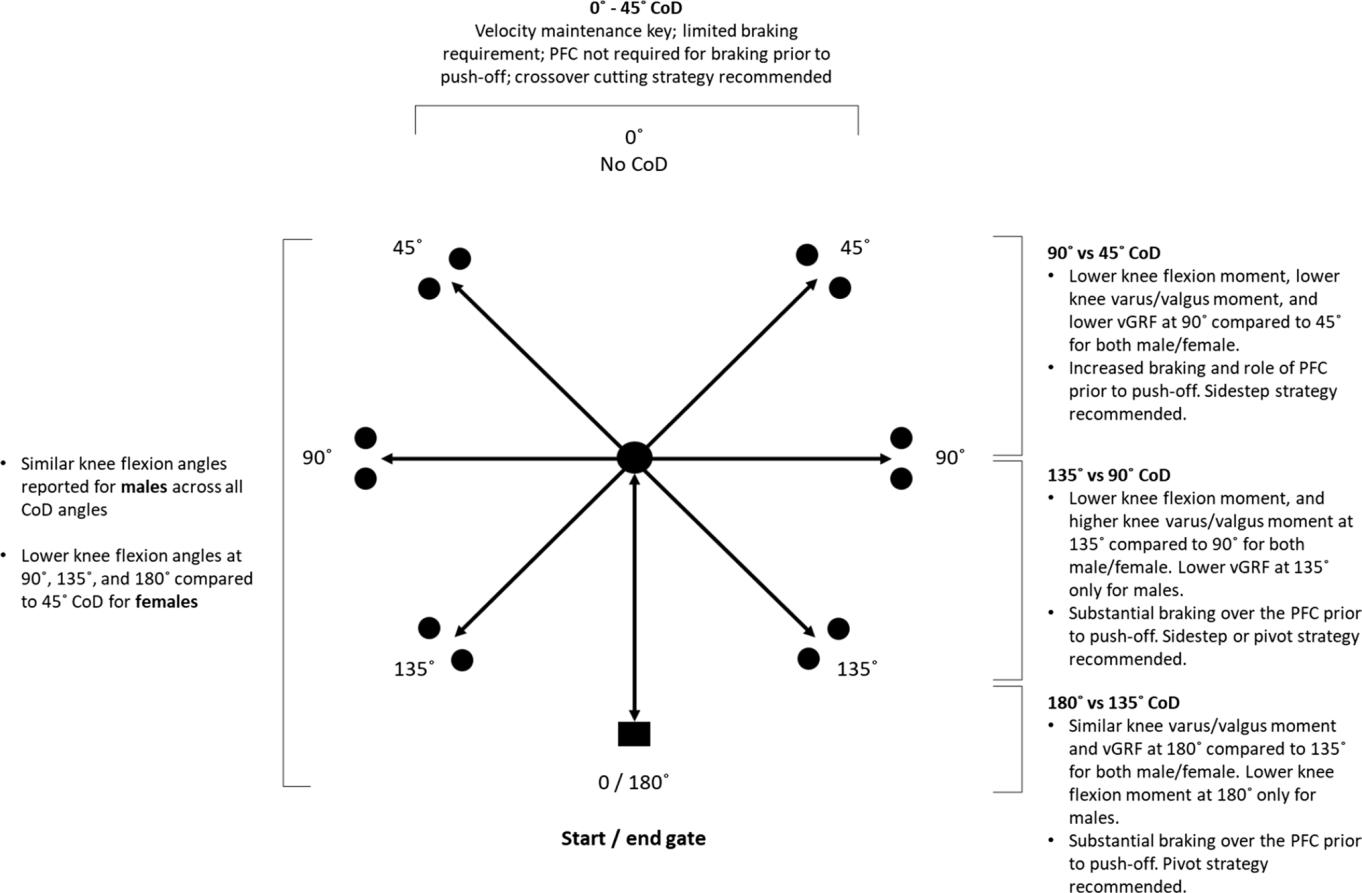
Kinetic and kinematic characteristics for males and females across different CoD angles (adapted from Dos’Santos et al. [23]; Schreurs et al. [77])

The magnitude of the load placed on the knee joint will be largely determined by the CoD angle required to perform the test [77]. A 45° cutting task has commonly been used to assess knee function following ACLr [78, 80] yet there is a heightened injury risk (i.e., knee valgus moment) increase at sharper cutting angles such as 90°, 135° and 180° turning when compared to 45° (Fig. 2) [77]. If approach velocity and stopping distance remain consistent across different cutting/turning angles, sharper CoD angles (≥ 90°) require an athlete to momentarily stop (to reach zero velocity) and brake harder. This increases the task demands as higher ground reaction forces are produced in shorter durations which in turn increases external loading on the knee joint and potential increase risk of injury [51]. In addition to changes in joint loading, the direction of force application during a CoD test is also highly dependent of the cutting angle. Sigward et al. [71] showed that the vertical, posterior and lateral ground reaction forces were 21%, 87% and 228% greater, respectively, during 110° cutting task in comparison with a 45° angle for both male and female soccer players. These data indicate that greater posterior and laterally directed forces are present for sharper CoD angles.

### Approach Velocity

CoD mechanics have also been shown to vary according to the approach velocity during the task, whereby athletes will self-organize their approach speed and entry velocity dependent upon the angle [23]. While not the case in all incidences, it appears non-contact ACL injuries occur more frequently during CoDs performed from higher approach velocities in multidirectional sports [53, 64]. Variables associated with ACL injury (i.e., knee flexion angles, knee valgus loading) increases during 45°, 60° and 135° cutting tasks from faster running velocities (4 and 5 m s^−1^) compared to slower velocities (2 and 3 m s^−1^) [40, 58, 82]. Thus, when interpreting the available literature, practitioners should be cognizant of these differences and how they affect joint loading. Athletes returning to sport following ACLr may also intentionally reduce their approach velocity if they perceive the demands of the CoD task are too great relative to their current capacity to decelerate effectively. Therefore, practitioners need to understand the influence and impact of cutting angle and approach velocity on join loading and CoD task demands in order to develop the most appropriate assessment protocols to assess knee function at different stages of rehabilitation following ACLr. In addition, allowing an athlete to self-select their approach and entry speed during testing (while potentially increasing the variability) will enhance the ecological validity and provide information as to their CoD strategy which can be monitored during rehabilitation to determine changes in performance and readiness to return to sport.

### Technique

CoD technique (kinematics) and the resultant load (kinetics) appear to be dependent on the task demands (e.g., angle, speed and mode) [24]. Athletes typically use three primary CoD techniques: the side-step, crossover cut, and split-step which display differences in execution and resultant mechanics [24]. For example, side-step has been considered as a key technique to feint an opponent, particularly during situations that required sharper CoD; however, it generates larger knee valgus and internal rotation moments compared to crossover cut and split-step techniques, suggesting higher risk of ACL injury [24]. The crossover cut technique on the other hand is commonly adopted by athletes when shallow CoD under high approach velocity is required to evade a defensive line or to pass by an opponent. Contrarily to side-step technique, crossover cut generates higher knee varus moment and loads the lateral component of the knee [24]. Finally, split-step can generate greater lateral velocity compared to side-step and crossover and hence, opponents find it as a difficult technique to be anticipated. Despite the lower knee abduction load displayed during split-step, this technique elicits longer ground contact time to be executed and as a result, it has been considered as a slower strategy for CoD compared to side-step and crossover techniques [24].

Biomechanical and technique differences have been also reported during extreme CoD techniques such as 180° turn compared to shallower cutting angles with athletes adopting different strategies due to the task constraints. Cortes et al. [16] showed that lower knee flexion (− 41.2 ± 8.8° vs. − 53.9 ± 9.4°) and a higher valgus angle (− 7.6 ± 10.1° vs. − 2.9 ± 10.0°) was present during 180° CoD task vs. the side-step (45°) and, respectively, at the point of maximum vertical ground reaction force. The 180° CoD task also had higher peak posterior ground reaction force than the drop-jump (0.8 ± 0.3 multiples of body weight vs. 0.3 ± 0.06 multiples of body weight) and side-step cutting (0.3 ± 0.1 multiples of body weight). In addition, higher internal varus moments were indicated in the 180° CoD task (0.72 ± 0.3 N m/kg m) as opposed to the drop-jump (0.14 ± 0.07 N m/kg m) and side-step (0.17 ± 0.5 N m/kg m) at peak stance.

According to Condello et al. [15], a more rounded shape strategy has been observed when athletes decrease contact time during the final step of a 60° cutting task. Team sport athletes usually adopt a sharper cutting strategy to effectively evade an opponent during competition. Thus, decreasing the contact time during the final step may be also detrimental for sports performance. The management of this performance–injury conflict is key to prepare athletes to RTS participation following ACLr. Practitioners should therefore, consider such important information to adopt adequate training strategies during the rehabilitation program.

### Visual Disturbance

Research has shown that experimentally visually cued temporal constraints can affect whole-body kinematics and knee loading during athletic activities such as cutting [3]. During side-cut conditions while attending to a ball, internal knee adductor moment was 20% greater (*p* = 0.03) and peak knee flexion angle was 4° larger (*p* < 0.01), compared to without the ball. Bjornaraa et al. [10] examined whether subjects with ACLr display different displacement, velocity, and time to peak ground reaction force during cutting activities than healthy subjects and whether visual disruption alters these variables. Knee displacement was significantly less for ACLr than non-dominant. Knee velocity was also significantly slower for ACLr vs. non-dominant limb with longer time to reach peak GRF. The authors reported a lack of vision resulted in reduced absolute velocity and displacement, especially for subjects with a history of ACL reconstruction; thus, suggesting the use of a new or altered ‘default’ motor program. Lieberman and Hoffman [50] observed that subjects with more experience in a task respond with less difference in movement patterns when vision is disrupted. Therefore, one could argue that environments should be as realistic and context-specific as possible when evaluating the ability to RTS. In addition, repeated exposures to these environments should form an important component of end stage rehabilitation to more fully prepare athletes for the demands of their sport.

### Dual Cognitive Task

During CoD tasks performed in competitive situations the cognitive load is increased since the athlete must allocate attentional resources to manipulate the ball and must also be aware of the location of their opponents, teammates and the goal and this in part may contribute to the higher risk of ACL injury during such movements [3, 74]. Negahban et al. [59] examined the effects of attention demands on postural control in ACLr patients who had return to sport > 12 months after surgery. Patients were required to perform a single-leg stance on a balance board under both single- and dual-task conditions in four dynamic balance tests. The authors showed that ACLr patients displayed higher contact frequency and longer contact time compared to healthy match controls. But more interestingly, ACLr patients showed higher contact frequency and longer contact time during dual-task compared to single-task conditions [59]. These findings highlight that concurrent execution of both postural and cognitive tasks led to performance deterioration of postural stability measures rather than cognitive measures. This suggests that the maintenance of standing balance is more attention demanding for ACLr patients than healthy matched controls. Therefore, it could be inferred that subtle alterations to testing protocols such as having participants attending to a ball [26], a defender [55], a simulated teammate [3], or simultaneous execution of a cognitive task [59] may have prominent effect on an athletes’ movement during CoD. As a result, the attentional demands of a task need to be carefully considered if the goal is to develop/implement an ecologically valid testing protocol. It is likely that practitioners may gain additional insight into the mechanics that contribute to non-contact ACL injury if they incorporate testing protocols that are reflective of the cognitive demands of the sports environment; however, due to the paucity of data and likely reductions in experimental control, this requires further investigation.

### Double Stimulation

Current COD test are broadly reflective of discrete aspects of athlete movement. Indeed, most COD assessments are either planned with one or few CoD or basic reactive decision responses (unplanned). This is very different to the complex decision-making process of one, or several, additional actions performed during matches. For example, this may involve two closely spaced movements (stimuli) by an attacking player performing a fake ball pass [31, 75]. The basis of the feint is the double-stimulation paradigm, where the reaction to the first of two closely spaced stimuli is normal, but the reaction to the second is delayed by more than that which would have occurred had it been presented alone [76]. Current CoD assessment methods are unable to examine these aspects. Achieving suitable control with acceptable test re-test reliability of such factors which occur in a chaotic environment is a challenging and possibly unrealistic ideal. Therefore, practitioners are encouraged to explore ways in which athletes can be exposed to these stimuli in training, with tasks clearly designed with different levels of contextual interference that are reflective of the athletes’ stage of rehabilitation and level of skill challenge required.

## Interpreting the Results

### Task Completion Time vs. Movement Execution

Time to complete the task has often been the sole marker for CoD assessment. Recently, King et al. [41] highlighted the limitations of solely using time when they identified biomechanical deficits when cutting using the involved vs. un-involved limb during a 90° cutting task despite no differences in performance time. In addition, Kyritsis et al. [44] used a cut-off of ‘pass score’ of < 11 s during a *T* test protocol as one of the criterion to discharge professional athletes back to sports participation. Munro et al. [54] reported mean performance values of 10.7 s for the same testing protocol performed by recreational university athletes who would be expected to have lower physical capacities. It is the opinion of the authors that CoD assessments should always have a task performance component but this should not be the only criteria due to the need to examine potential deficits in lower extremity biomechanics which may contribute to a greater risk of re-injury [78]. Using total time solely to measure CoD is not sufficient to identify important qualitative information (e.g., trunk position, foot placement, center of mass height, knee angles, arm actions and visual focus) presented by an athlete while executing the CoD movement. In addition, practitioners are advised to utilize performance cut-off ‘pass scores’ that represent the high performances required by elite athletes returning to professional sport if that is the intended destination. Finally, wherever possible, general cut-off ‘pass scores’ should be replaced by individual pre-injury performance data to make decisions relative to the individual (considering differences in strength, speed, CoD ability, etc.). Applying the same absolute score to all athletes could be too conservative/demanding for faster and slower athletes, respectively, highlighting the importance of collecting base-line data on all players as part of a regular screening/monitoring program.

### The Importance of Deceleration

Deceleration is a fundamental component of multidirectional speed to allow athletes to effectively change their state of momentum. Rapid deceleration (stop type activities) are a consideration for ACL injury due to an increase in anterior tibial shear force and anterior tibial translation [70]. Despite deceleration is present in all sprinting and CoD tasks, they have often been investigated individually. Peel et al. [66] examined lower extremity biomechanics between both decelerating and 45° cutting tasks as well as the relationship between peak anterior shear force (ASF) and peak knee abduction moments (KAM) during both decelerating and cutting tasks. It has been found that the CoD task exhibited significantly higher initial contact knee abduction angles (*p* = 0.032, *d* = 0.62), higher KAM (*p* ≤ 0.001, *d* = 2.84) and larger ASF (*p* = 0.001, *d* = 1.42) compared to the decelerating task. This suggests that CoD induces higher mechanical stress at the knee joint and hence, an increased risk of ACL injury compared to decelerating task. The authors also reported a positive relationship (*p* = 0.67) for ASF during both decelerating and CoD tasks. However, a negative relationship (*p* = − 0.21) was found between both tasks for KAM, suggesting that decelerating and cutting are independent of one another. In addition, it has been observed a negative correlation (*p* = − 0.43) between ASF and KAM during both decelerating and CoD tasks. While peak ASF increases, peak KAM decreases, indicating that these two variables are autonomous [66]. Due to these distinct differences, it could be suggested that measurement of deceleration as an isolated construct (without the CoD component) may be an appropriate assessment which can be used at an earlier stage of on-pitch/court rehabilitation to examine an individual’s ability to effectively apply braking forces as a pre-cursor to CoD.

In support of the need to examine an athlete’s approach to deceleration, the role of the preparation steps prior to turning or cutting should be considered. Higher cutting angles (i.e., ≥ 60°) require greater reductions in velocity to change the athletes state of momentum [27–29]. Greater braking forces during the penultimate foot contact ensure that athletes can maintain a higher entry velocity (as they can brake later) which results in faster CoD speed, and reduce the ground reaction force on the turning limb during the plant step [36]. This has important connotations as non-contact ACL injuries often occur on the planted limb during a sudden deceleration prior to a change in direction [8]. Maintenance of foot alignment in the direction of travel during the penultimate step ensures the quadriceps are in an advantageous position. This technical model is more effective and potentially ‘safer’, whereby, the production of the highest magnitude of braking forces required to effectively decelerate takes place in the penultimate, as opposed to the turn step, when the foot has already laterally rotated and is in a more ‘vulnerable position’. Thus, it may be prudent to examine kinematics and loading characteristics during the penultimate step to more fully understand the athlete’s deceleration strategy. Currently, methods to assess these qualities on the field/court are sparse; thus, further research is warranted using practically viable testing protocols.

### Limb Symmetry

As previously indicated, biomechanical differences between involved (ACLr limb) and uninvolved limb were found during both planned and unplanned CoD tasks despite no differences in task performance [41]. Myer et al. [56] also did not identify performance differences when assessing the *T* test performance favoring either the involved or un-involved limb. These data suggest that athletes develop compensatory movement strategies to complete the task with similar performance levels. This finding questions whether returning an involved limb to the standard of the uninvolved limb should be considered as the gold standard during tasks such as CoD. It is also unclear if the current level of performance on the uninvolved limb is representative of their pre-injury capacity [86]. Furthermore, Thomas et al. [81] reported that athletes adopt different braking strategies between dominant and non-dominant limbs during a 180° turn, whereby, a greater magnitude of horizontal braking forces is placed on the penultimate step by the non-dominant limb when turning with the dominant limb. Conversely, a greater emphasis is placed on the final foot contact when turning with non-dominant leg [81]. Thus, limb symmetry is just one component of the return to sport puzzle and assessment of movement characteristics during CoD should be considered alongside the demands of the sport, estimated pre-injury capacity, preferred limb dominance and potential risk for re-injury.

## Practical Applications

**Fig. 3:**
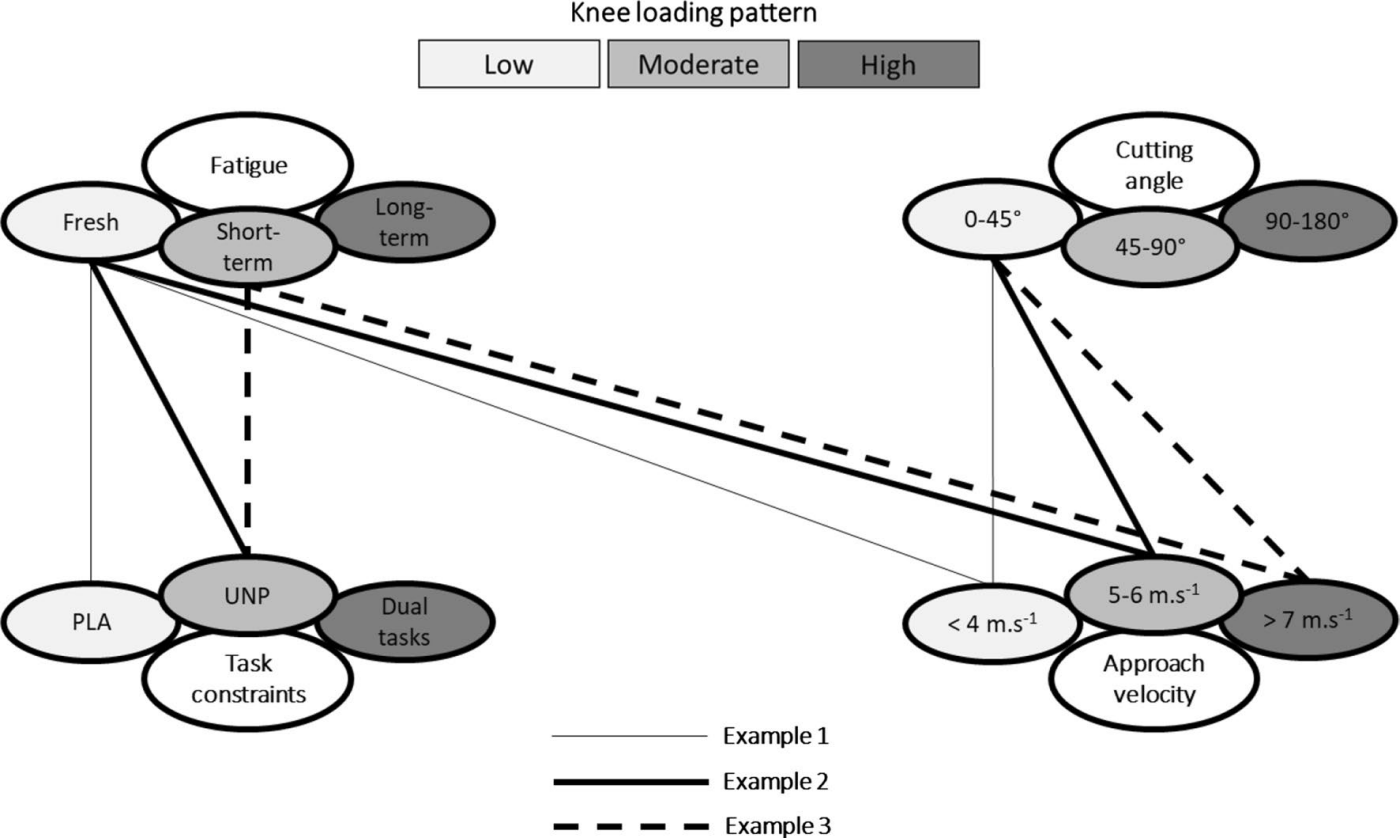
The model displays an illustrative example of a CoD assessment progression sequence to support practitioners in setting up effective CoD test prescription to assess knee function following ACLr. The figure demonstrates how key testing conditions/constraints can be manipulated to increase knee loading progressively during a 45° CoD task across the rehabilitation process. Example 1 requires low approach velocity (< 4 m s^−1^), in a non-fatigue state, under planned conditions. This approach induces low stress at the knee joint and can be performed at the beginning of the functional phase of rehabilitation in order to allow practitioners to identify potential technical issues (i.e., foot placement) while executing the CoD task. Example 2 is a progression and includes higher approach velocity (5–6 m s^−1^) and a constraint during the CoD task (e.g., responding to an external stimulus). Finally, example 3 represents an assessment design that induces higher stress at the knee joint and hence, its implementation may be more appropriate during an advanced phase of the rehabilitation process (i.e., sport-specific phase). It requires high approach velocity (> 7 m s^−1^), followed by a short-term fatigue testing protocol (e.g., repeated sprints), under unplanned conditions. This approach may allow practitioners to identify progressions in movement execution in relation to previous assessments, as well as the ability of the patient to maintain appropriate mechanics during the CoD task while fatigued

Based on the aforementioned considerations, a CoD assessment model is illustrated in Figure 3. While not designed to be prescriptive, the information provides examples of a progression and regression sequence of four-fundamental aspects discussed within the current review (i.e., angle, speed, fatigue and task constraint). The aim of this approach is to illustrate how key test conditions/constraints can be manipulated for effective prescription of CoD assessment (and training) at different stages of rehabilitation following ACLr. This model may help practitioners to establish appropriate CoD testing protocols in agreement with the load athletes are able to tolerate based on their level of function and stage of rehabilitation.

For example, exposing athletes who are returning to sport following ACLr to sharper CoD angles requires an abducted lower extremity position, leading to greater external loading on the knee joint and subsequent higher risk of re-injury [51]. However, this approach is necessary to create a sufficient lateral foot plant distance to apply effective propulsive forces and execute the task with maximal performance [33, 35]. Since sharper cutting angles and faster changes in direction are determinants for successful performance in multidirectional and invasion sports [11, 68], practitioners should be aware of this performance–injury conflict. A lack of exposure to these more extreme changes of direction may leave the athletes underprepared for the demands of match play, leading to an increased risk of re-injury. Conversely, shallower cutting angles display lower joint loading and may be more appropriate for those in the earlier phases of their rehabilitation.

Another example of how these considerations can also be shown using the concept of approach velocity. Low approach velocities will reduce knee joint loading; however, it will not prepare them for sport-specific scenarios. In professional sport, athletes are unlikely to sacrifice performance at the expense of reduced knee joint loading [23]. Therefore, this performance–injury conflict should once again be managed during the rehabilitation process. Prior to exposing ACLr patients to tasks at maximal approach velocities during a CoD test, practitioners should ensure that patients display appropriate CoD mechanics [20, 21, 35, 45] and the requisite physical capacities to tolerate the associative knee joint loading [37, 63, 65, 72, 83]. Furthermore, approach velocities (and distances) should be gradually increased as the athlete progress and moves closer to return to sport. Specifically, monitoring of approach velocity during maximal CoD tasks could also be a practically viable approach and may be linked to confidence as well as physical and technical competence. Furthermore, integration of other factors discussed within this review, such as double stimulation and visual disturbance can be utilized where appropriate to create heightened contextual interference in the later stages of rehabilitation as a means of increasing the cognitive overload of CoD tasks.

## Conclusion

CoD assessments have not been widely implemented by practitioners as a criterion measure to inform RTS decision-making. Furthermore, current field-based practices do not appear in agreement with relevant sport demands, or effective in their assessment of knee function following ACLr. Laboratory-based studies have shown residual deficits in movement mechanics in athletes at the time of return to sport, and this in part may be related to risk of re-injury. However, these assessments have shown several limitations and are likely not practically viable for monitoring progress during rehabilitation.

In this review we have detailed a number of factors for the development of future practice in the area of CoD assessment following ACLr and provided suggestions for how these can be incorporated. Fundamentally, individual risk factors for the athlete and their sport should be examined to determine their state of readiness to RTS. In doing so, it is essential that clinicians understand the different layers of CoD assessment; most importantly, the conditions/constraints under which they are performed. Analysis of movement mechanics is also an important focus; as total time alone is likely insufficient to accurately characterize performance decrements. To do this, practically viable solutions for on-pitch/court measurement are now needed to allow coaches to ‘bridge the gap’ between the laboratory and the sports environment. This approach may facilitate a more informed decision-making process with the end goal being, a ‘return to performance’ with a lower risk of re-injury.
